# Carbon Nanotube Flexible and Stretchable Electronics

**DOI:** 10.1186/s11671-015-1013-1

**Published:** 2015-08-12

**Authors:** Le Cai, Chuan Wang

**Affiliations:** Department of Electrical and Computing Engineering, Michigan State University, East Lansing, MI 48824 USA

**Keywords:** Carbon nanotube network, Thin-film transistor, Flexible electronics, Stretchable electronics, Printed electronics

## Abstract

The low-cost and large-area manufacturing of flexible and stretchable electronics using printing processes could radically change people’s perspectives on electronics and substantially expand the spectrum of potential applications. Examples range from personalized wearable electronics to large-area smart wallpapers and from interactive bio-inspired robots to implantable health/medical apparatus. Owing to its one-dimensional structure and superior electrical property, carbon nanotube is one of the most promising material platforms for flexible and stretchable electronics. Here in this paper, we review the recent progress in this field. Applications of single-wall carbon nanotube networks as channel semiconductor in flexible thin-film transistors and integrated circuits, as stretchable conductors in various sensors, and as channel material in stretchable transistors will be discussed. Lastly, state-of-the-art advancement on printing process, which is ideal for large-scale fabrication of flexible and stretchable electronics, will also be reviewed in detail.

## Review

Unlike conventional microelectronics/nanoelectronics, whose emphasis is miniaturization for ultimate performance and integration density, macroelectronics focuses on large-area and low-cost applications and new form factors such as flexible and stretchable devices [1]. Electronic devices that are fabricated on plastic or rubbery substrates such as flexible display, electronic paper, smart packages, skin-like sensors, wearable electronics, implantable medical implements, and many others could radically change people’s perspectives on electronics [2]. The development of those new forms of electronics relies largely on the advancements in material science. Over decades, amorphous silicon, polysilicon, and organic semiconductors have been extensively studied as the channel materials for thin-film transistors (TFTs), one of the key components in macroelectronics [3–6]. In recent years, nanomaterials, including quantum dots [7], one-dimensional carbon nanotubes and nanowires [8, 9], as well as two-dimensional (2D) materials [10, 11] have attracted numerous research interests in this area because they offer significantly better performance than organic semiconductors and are easier to process than a-Si or polysilicon. In particular, carbon nanotubes (CNTs) hold great promise for high-performance flexible electronics due to their extremely high carrier mobility, superior mechanical flexibility, and stability [12].

A single-wall carbon nanotube (SWCNT) can be considered as a seamless cylinder formed by rolling up a graphene sheet along a vector *C*_*h*_ = n***a***_***1***_ + m***a***_***2***_, where ***a***_***1***_ and ***a***_***2***_ are the basis vectors of the hexagonal crystal lattice of graphene. The indices (*n*, *m*) define the two structural parameters, diameter and chirality, of the nanotube. Theoretical calculations indicate that, depending on the indices, the nanotube can have different electrical attributes—metallic for *n*-*m* equals multiples of 3, and semiconducting for others. In addition, the bandgap of a semiconducting nanotube is known to be inversely proportional to its diameter [13].

The unique structure-property relation makes SWCNTs ideal candidates for molecular electronic devices—e.g., metallic nanotubes act as interconnects while semiconducting SWCNTs play the role of channel material for field-effect transistors (FETs) [14]. Additionally, numerous studies have already revealed that individual SWCNTs exhibit very exciting electronic properties, which are well beyond their conventional material counterparts. For instance, the current carrying capability of metallic SWCNTs can reach 10^9^ A/cm^2^ (much better than aluminum or copper) while semiconducting SWCNTs can exhibit field-effect mobilities up to 10^4^ cm^2^V^−1^ s^−1^ (far exceeding silicon) [15–17]. Nevertheless, devices based on individual SWCNTs suffer from poor uniformity and reproducibility, mainly due to difficulties in reliable synthesis of SWCNTs with homogeneous structural attributes, as well as controllable assembly of SWCNTs over a large area [12, 18]. In addition, novel fabrication methods are needed to render the individual-SWCNT-based devices compatible with current industrial manufacturing processes. On the other hand, macroscale assemblies of SWCNTs, particularly random networks and thin-films, are believed to enable the most realistic applications of SWCNTs in electronics in the short term because they not only offer facile processing but also uniform and reproducible performance due to ensemble averaging [12, 19, 20]. Additionally, SWCNT networks are especially suitable for flexible and stretchable electronics because the lateral deformation of the curvy and entangled SWCNTs can accommodate practically large strains [21, 22]. In fact, there have already been lots of studies demonstrating the great promise of SWCNT networks as the channel materials and/or electrodes in various types of flexible/stretchable electronic devices, such as integrated circuits [23–28], sensors [22, 29–31], organic light-emitting diodes (OLEDs) [32, 33], supercapacitors [34–36], touch panels [37], and so on.

**Fig. 1:**
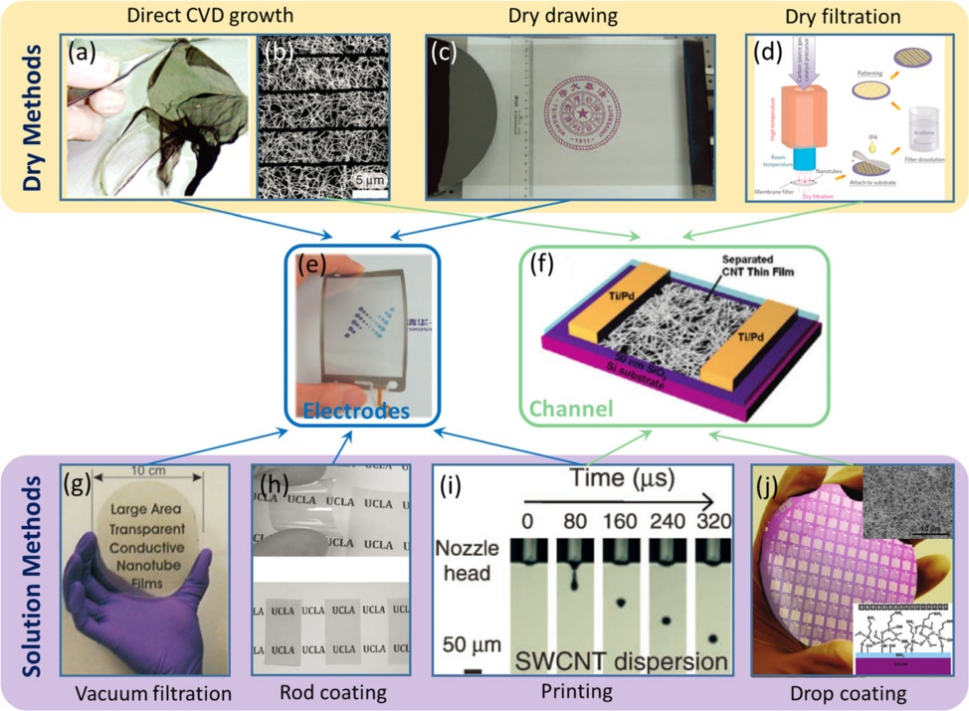
Various methods for preparing carbon nanotube networks and representative electronic applications. Dry process (**a**-**d**): **a** Photograph of directly CVD grown freestanding SWCNT thin film. Reproduced from ref. [38]. **b** SEM image of CVD-grown SWCNT network patterned into strips for channel materials of flexible TFTs. Reproduced from ref. [24]. **c** Photograph of multi-wall carbon nanotube (MWCNT) thin films obtained using a dry drawing process. Reproduced from ref. [40]. **d** Schematics of dry filtration process to collect sparse SWCNT network for flexible TFTs. Reproduced from ref. [25]. **e**, **f** Applications of carbon nanotube networks as flexible electrodes (**e**) (reproduced from ref. [37]) or channel materials for flexible TFTs (**f**) (reproduced from ref. [44]). Solution process (**g**-**j**): **g** Photograph of SWCNT thin film produced by vacuum filtration. Reproduced from ref. [42]. **h** Photographs of rod-coated SWCNT films with different transmittance. Reproduced from ref. [33]. **i** Time-dependent snapshot of SWCNT ink droplet during ink-jet printing process. Reproduced from ref. [45]. **j** Photograph of wafer-scale SWCNT TFTs fabricated using drop-coated SWCNT network. Insets: SEM image of the SWCNT network and schematic illustration of the surface chemistry used. Reproduced from ref. [44]

Several strategies are currently available to prepare CNT networks and thin films as illustrated Fig. 1. Generally, they can be classified in two categories: dry processes and solution processes [19, 20]. Dry processes are mainly direct chemical vapor deposition (CVD) growth and dry drawing from vertically aligned CNT arrays [8]. Direct CVD-grown SWCNT films comprise ultralong nanotubes bonded by strong interbundle connections [38] and thereby possess excellent conductivity, making them suitable for the electrode material of many functional devices like super-fast actuators [39], stretchable supercapacitors [34–36], and strain sensors [31]. However, the size of CVD furnaces limit the area of this kind of SWCNT films to typically below 100 cm^2^ [38]. Although dry drawing method can, in principle, continuously produce CNT films with unlimited area [40, 41], the direct drawing of SWCNTs from vertical arrays has yet to be realized. Moreover, since there is currently no effective method to grow structurally or electrically homogeneous SWCNTs, the biggest disadvantage of CVD-grown SWCNT films is that the as-grown samples typically contain a mixture of CNTs with all types of chirality and metallicity. Therefore, such samples cannot be used as channel materials for transistors until the metallic conduction is eliminated by special process such as electrical breakdown, stripe patterning, or dry filtration [24, 25].

On the other end of the spectrum lies the solution-based process, where several methods have been reported including vacuum filtration [42], rod coating [43], drop coating [26, 44], and printing [45–47]. The solution process of CNTs is enabled by successfully dissolving them in suitable organic solvents or in aqueous solution with the assistance of certain types of surfactants [48, 49]. SWCNT thin films obtained by vacuum filtration and rod coating have been used for flexible, stretchable, and transparent electrodes [33, 50], while printed SWCNT networks have been demonstrated to act as both electrodes and channel materials for TFTs [45]. One key advantage of solution-based process is low temperature and compatibility with various types of widely used polymeric materials, thus enabling the low-cost and large-scale deposition onto various flexible and stretchable substrates [12, 20]. More importantly, through the solution process, it is possible to selectively assemble the SWCNTs with the same electronic type or chirality obtained by post-growth purification and separation [51–53]. With the tremendous progress in SWCNT separation and purification, semiconductor-enriched SWCNTs (sSWCNTs) are now available in large quantities [54], which enables the wafer-scale fabrication of sSWCNT TFTs with high yield and uniform performance [44]. The most significant advantage of using sSWCNT networks for TFT application lies in the unique combination of superior flexibility/stretchability, optical transparency, and low-temperature solution process, which are not possible with conventional polysilicon or amorphous silicon platforms [3, 4]. In addition, compared with organic semiconductors, sSWCNT networks not only offer drastically better air stability but also multiple orders of magnitude improvements in carrier mobility [5].

The realization of large area flexible/stretchable electronics also relies on the innovations in manufacturing techniques. Conventional microfabrication processes used in semiconductor industry is not desirable due to its high cost, limitation in sample size, and restrictions in substrate material. Alternatively, printing is a promising method with theoretically no restrictions in substrate material and size [55, 56]. In addition, as an additive process, printing produces minimum material waste and thereby enables eco-friendly and cost-effective manufacturing. Recently, several groups have reported printed flexible devices and circuits using solution-processed SWCNTs, representing a viable way to large-scale and low-cost flexible electronics based on SWCNTs [45–47, 57–66].

In this paper, we survey the recent progress on flexible and stretchable electronics with SWCNT networks as either electrodes or channel materials. This review is organized as follows. In “Carbon Nanotube Networks for Applications in Flexible Electronics” section, we briefly discuss progress made on the high-performance flexible electronics with sSWCNT networks as channel semiconductors. Herein, three examples are presented, namely, flexible TFTs, integrated circuits, and electronic skins. For more detailed and systematic discussion of sSWCNT-based flexible electronics, readers are referred to the recent review papers covering this topic [12, 67]. In “Carbon Nanotube Networks for Applications in Stretchable Electronics” section, we highlight several applications of SWCNT networks in stretchable electronics, including stretchable conductors and electrodes, sensors, and TFTs. In “Scalable Fabrication Process—Printing” section, we focus on the recently developed fabrication process that is most suitable for large-area flexible/stretchable electronics—printing. The state-of-the-art development of fully printed SWCNT-based TFTs and integrated circuits are discussed in detail. Lastly in “Conclusions” section, we conclude with the current remaining challenges and future prospects in this area.

### Carbon Nanotube Networks for Applications in Flexible Electronics

**Fig. 2:**
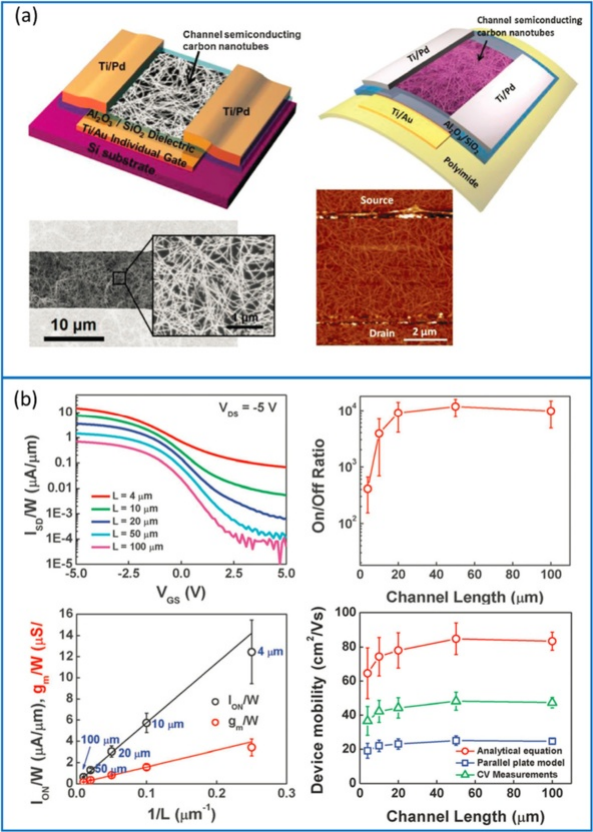
High-performance TFTs using semiconducting carbon nanotubes (sSWCNTs). **a** Schematics of TFTs on rigid (*upper left*) and flexible (*upper right*) substrates. The SEM (*lower left*) and AFM (*lower right*) images of the channel region are also shown. **b** Electrical characteristics of the flexible TFTs. *Upper left*: *I*_d_-*V*_g_ curves of transistors with different channel lengths. *Upper right*: TFT on/off current ratio as a function of channel length. *Lower left*: normalized on-current and transconductance as functions of reciprocal channel length. *Lower right*: field-effect mobility, calculated based on C-V measurement (*green*), as a function of channel length. Also shown are the mobility obtained using parallel plate model (*blue*) and cylindrical model (*red*). Reproduced from ref. [12]

**Fig. 3:**
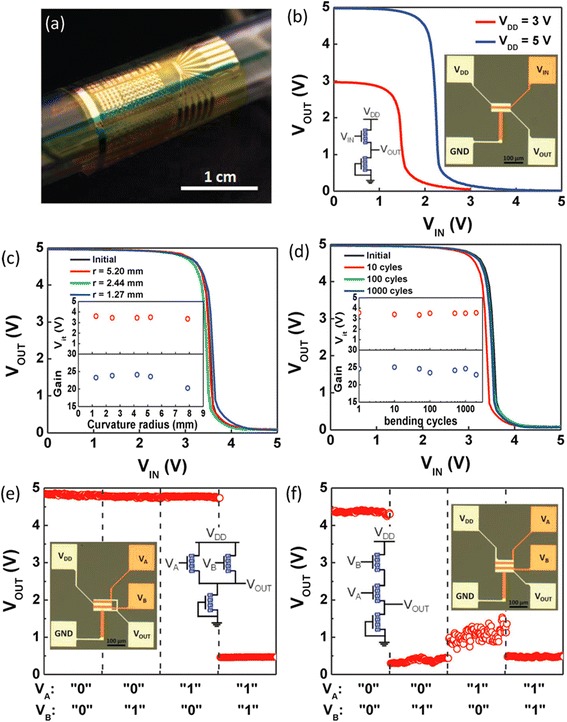
Flexible logic gates using CNT-TFTs. **a** Optical image demonstrating the flexibility of the circuits, where the sample is wrapped onto a glass tube with a diameter of 10 mm. **b** Inverter voltage transfer characteristics (VTC) measured with a V_DD_ of 3 or 5 V. Inset shows the schematic and the optical picture of the diode-loaded inverter. **c** Inverter VTC measured while the circuit is bent to various curvature radii. Inset shows the inverter threshold voltage and gain as a function of curvature radius. **d** Inverter durability under cyclic bending test, showing stable performance for up to 2000 cycles. **e, f** Output characteristics of diode-loaded 2-input NOR (**e**) and NAND (**f**) gates. *V*_DD_ is 5 V for both circuits. Insets are the corresponding schematic and optical micrographs. Reproduced from ref. [26]

TFT plays a critical role in macroelectronics. As discussed previously, semiconductor-enriched single-wall carbon nanotubes (sSWCNTs) are ideal candidates for the channel material of flexible TFTs because of the unique combination of low-temperature processing, mechanical compliance, optical transparency, and superior electrical property. Using high-purity sSWCNT solutions, Wang et al*.* obtained wafer-scale nanotube networks with high density and uniformity [44], which subsequently enabled the fabrication of TFTs and logical circuits on both rigid and flexible substrates [26]. With the assistance of suitable surface chemistry and by controlling the sSWCNT deposition time, the network density can be fine adjusted (as high as 65 tubes/μm^2^), which in turn determines the ultimate device performance (Fig. 2a). Flexible TFTs fabricated on such sSWCNT networks exhibit p-type conduction with on/off current ratio (*I*_on_/*I*_off_) on the order of 10^4^ when the channel length is above 10 μm (Fig. 2b). In devices with channel lengths of 4 μm, on-current (*I*_on_/*W*) and transconductance (*g*_m_/*W*) reach 15 μA/μm and 4 μs/μm, respectively, at a moderate voltage of 5 V. Due to the trade-off between on/off ratio and transconductance, TFTs with large channel lengths (high *I*_on_/*I*_off_) are ideal for logical circuits while the devices with short channel lengths (large *g*_m_) are suitable for analog and radio-frequency application. In addition, capacitance-voltage (C-V) measurements are performed to precisely determine the gate capacitance which in turn leads to an accurate assessment of the field-effect mobility (*μ*), with a typical value of ~50 cm^2^V^−1^ s^−1^ (Fig. 2b) [26], similar to that of low-temperature polysilicon (LTPS) and much higher than those of amorphous silicon and organic semiconductors. Furthermore, the use of ultrathin polyimide substrates results in highly flexible TFTs and integrated logical circuits, including inverter, NOR, and NAND gates. Such devices and circuits exhibit excellent stability after thousands of bending cycles with curvature radii down to ~1 mm (Fig. 3).

**Fig. 4:**
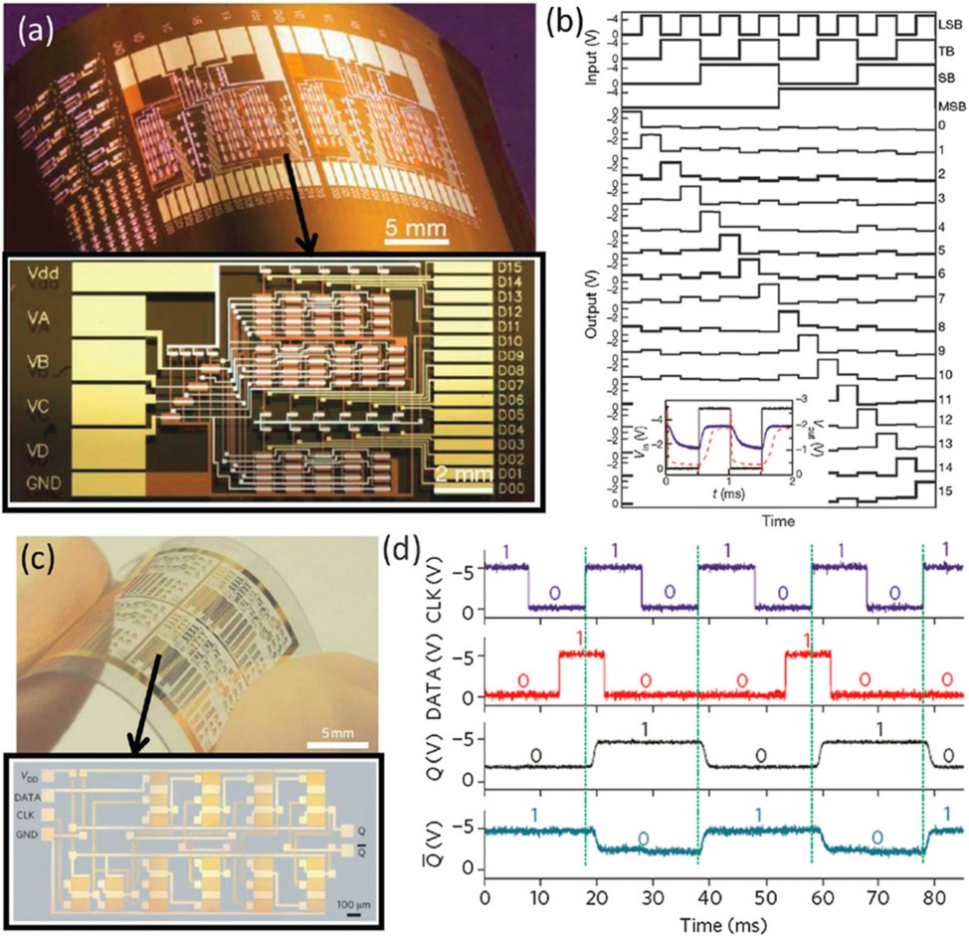
More sophisticated flexible integrated circuits using CNT-TFTs. **a** Optical image of a 4-to-16 decoder. **b** Transient response of the decoder. Reproduced from ref. [24]. **c** Optical images of a D-flip-flop. **d** Transient response of the D-flip-flop. Reproduced from ref. [25]

High-performance TFTs and integrated logical circuits have also been fabricated using random networks of single-wall carbon nanotubes directly grown from CVD methods. Compared with solution-process SWCNTs, CVD-grown SWCNTs often have longer tube length and cleaner surface, which are advantageous for obtaining better device mobility owing to fewer inter-tube junctions and less surface scattering. However, since roughly one-third of the as-grown SWCNTs are metallic, additional process are needed to suppress the metallic conduction within the SWCNTs network. Cao et al. adopt strip patterning to reduce the probability of percolative metallic pathways in the network and obtained transistors with *I*_on_/*I*_off_ as high as 10^5^ without significant sacrifice in carrier mobility (~80 cm^2^V^−1^ s^−1^) [24]. Medium-scale integrated circuits consisting of up to nearly 100 transistors were further demonstrated on plastic substrates (Fig. 4a, b). In another report, Sun et al. fabricate TFTs and integrated circuits on transparent and flexible substrates using sparse networks of SWCNTs that are collected from a CVD furnace with a dry filtration method [25]. The TFTs exhibit field-effect mobility and *I*_on_/*I*_off_ of 35 cm^2^V^−1^ s^−1^ and 10^6^, respectively (Fig. 4c, d). The high electrical performance benefits from the long nanotube lengths (~10 μm) as well as Y-type inter-nanotube junctions which have large contact area and thus low contact resistance.

**Fig. 5:**
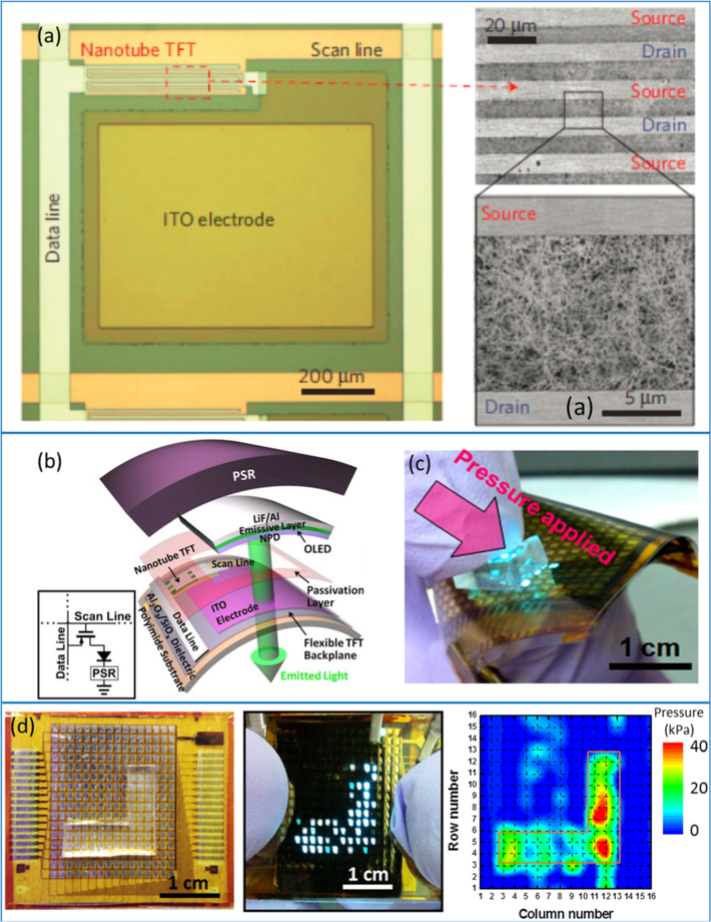
User-interactive electronic skin (E-skin) using CNT-TFTs. **a** *Left panel*: optical micrograph of one pixel before integrating OLED and pressure sensitive rubber (PSR). *Right panel*: SEM image showing the channel region of the CNT-TFTs. **b** Schematic diagram showing the exploded view of one pixel, consisting of a CNT-TFT, an OLED, and a layer of PSR vertically integrated on a polyimide substrate. **c** Operation of the E-skin system with 16 × 16 pixels, showing the OLEDs are turned locally on where pressure is applied. **d** Optical and electrical readout from the E-skin system when an L-shaped PDMS slab is used to apply pressure onto the sample. Reproduced from ref. [72]

In addition to digital integrated circuits, flexible carbon nanotube TFTs have also been incorporated into various functional systems, including sensors [68, 69], displays [70, 71], and electronic skins (E-skins) [72]. Figure 5 presents an example of user-interactive E-skin where an sSWCNT TFT array is used as the active matrix backplane for driving organic light-emitting diode (OLED) display and pressure-sensitive rubber (PSR) connected in series [72]. The pixel can be turned on locally where the surface is touched (Fig. 5c) and the light intensity of the OLED qualitatively represents the magnitude of the applied pressure, enabling both electrical readout and visual output of the external stimuli with a high spatial resolution (Fig. 5d). This example represents a system-on-plastic demonstration where three distinct electronic components—carbon nanotube TFT, OLED display, and pressure sensor—are monolithically integrated over large area on a single piece of flexible substrate. Substituting other sensors, such as chemical sensor, light sensor, and temperature sensor, for the pressure sensor used here could allow various functionalities akin or superior to natural skins and find a wide range of applications in smart robotics and security/health-monitoring devices.

### Carbon Nanotube Networks for Applications in Stretchable Electronics

Mechanical flexibility by itself may not be sufficient for some applications. For instance, a surface with nonzero Gaussian curvature like a sphere or an irregular surface like the elbow could never be conformally covered with a system that is only flexible [73]. Instead, stretchable electronics could fill in. One strategy to realize stretchable devices is based on thin films of conventional bulk semiconductors like Si and GaAs that are configured into wavy or buckling structures and bonded on elastomer substrates [74–76]. However, this buckling method is rather complicated to fabricate and may not be suitable for large area or mass production. Alternatively, systems that are intrinsically stretchable can be built by using organic materials or nanomaterials with relatively simple process [77].

Because of the extreme aspect ratio, carbon nanotubes are naturally highly curved and entangled in their macroscale assemblies, making them ideal materials for stretchable electronics [77, 78]. In situ scanning probe microscopic observations also reveal that, upon stretching and releasing process, carbon nanotubes form wavy structures, either in plane or out of plane [21, 22], that could accommodate further deformations. Generally speaking, metallic nanotubes can be used as stretchable interconnects and electrodes [22, 33–36, 79–85] while semiconducting nanotubes can take the role of channel materials for stretchable TFTs [27, 28, 68]. In the following section, we first discuss the carbon nanotube stretchable conductors and their applications and then shift focus to the stretchable transistors with semiconducting carbon nanotubes as channel materials.

**Fig. 6:**
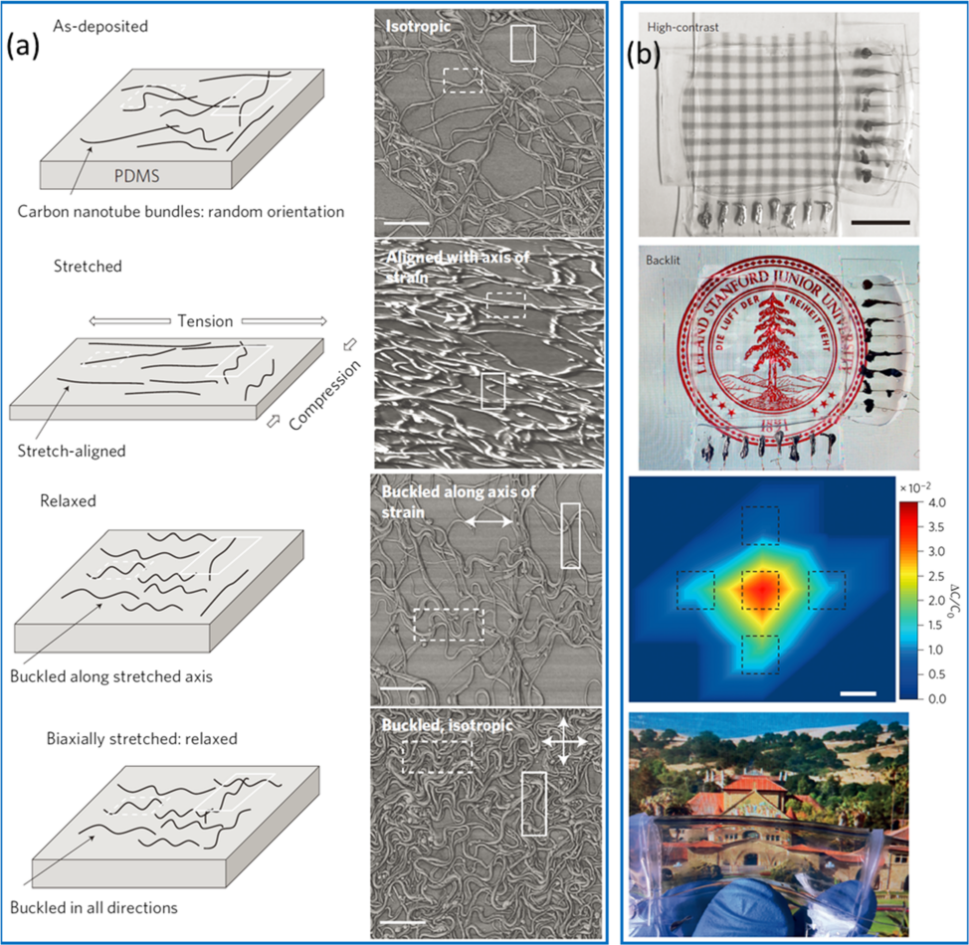
Carbon nanotube network as stretchable transparent electrodes for skin-like pressure sensor applications. **a** Schematics (*left*) and corresponding AFM images (*right*) showing the microscopic morphology evolution of CNT network on PDMS substrate when subjected to uniaxial and biaxial stretching and releasing tests. **b** Optical images and two-dimensional pressure profile obtained from a transparent pressure sensor array. Reproduced from ref. [22]

Thin films and networks of carbon nanotubes usually exhibit high optical transparency, which provides additional advantages for the applications as electrodes of stretchable electronic and optoelectronic devices [8, 20]. Lipomi et al*.* fabricated highly stretchable and optically transparent electrodes by spray coating SWCNT solutions onto polydimethylsiloxane (PDMS) substrates (Fig. 6) [22]. The resistance change of the SWCNT/PDMS stretchable conductors exhibits a unique strain-history-dependent behavior during the initial stretching-releasing tests with progressively increasing strains, implying the strain-dependent evolution of the microscale morphology of the SWCNT network. Systematic electromechanical characterizations reveal that the SWCNT/PDMS stretchable conductors can retain stable conductance after 10,000 cycles of stretching with strains up to 25 %. As an application, passive matrix of transparent capacitive sensor arrays with the capability of strain and pressure detection are further demonstrated by using the SWCNT/PDMS stretchable conductors as electrodes.

**Fig. 7:**
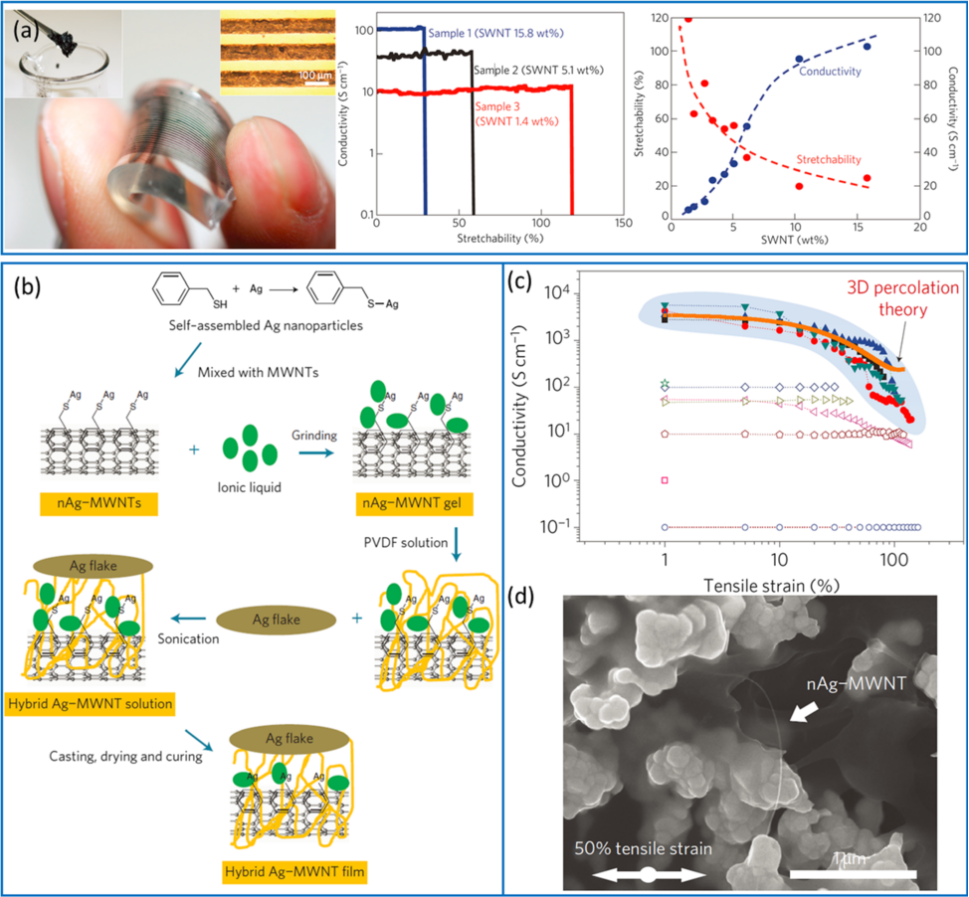
Stretchable conductors based on CNT-polymer composites. **a** Elastic conductors comprising super-growth SWCNTs uniformly dispersed in a fluorinated rubber: *Left*, optical images of screen printed patterns with a feature width of 100 μm; *middle*, conductivity as a function of tensile strain for three samples with different SWCNT content; *right*, stretchability and conductivity as a function of SWCNT content. Reproduced from ref. [80]. **b**-**d** Highly conductive and stretchable hybrid composites of silver flake and MWCNTs decorated with self-assembled silver nanoparticles. **b** Diagram of the synthesis process. **c** Conductivity as a function of tensile strain. **d** SEM images of the nanocomposites at a strain of 50 %. Reproduced from ref. [81]

Although carbon nanotube films embedded in elastomers are excellent materials for stretchable electrodes, they are not the best choice for stretchable interconnects that require not only good stretchability and conductivity but also easy processing using direct writing or printing. In this regard, the composites consisting of carbon nanotubes and suitable polymeric binders show great promise [78]. Large nanotube lengths and uniform dispersion are desired to obtain composites with good performance. Sekitani et al*.* fabricated printable SWCNT pastes by uniformly dispersing super-growth SWCNTs in a fluorinated rubber with the assistance of an ionic liquid and a high-pressure jet-milling process [79, 80]. Long and fine SWCNT bundles form well-developed conducting networks in the rubber matrix. As shown in Fig. 7a, fine features of SWCNT stretchable conductors can be patterned by screen printing. The conductivity and stretchability show inverse dependence on SWCNT content—higher SWCNT load leads to better conductivity but lower stretchability. The highest conductivity and stretchability achieved are 102 S/cm (15.8 wt% SWCNT) and 100 % (1.4 wt% SWCNT), respectively. Further improvement of conductivity can be achieved by adding metallic additives in the composite. Chun et al. synthesized hybrid composites consisting of micrometer- sized silver flakes and multi-wall carbon nanotubes (MWCNTs) decorated with self-assembled silver nanoparticles [81]. Silver nanoparticles (~3 nm) with phenyl rings are conjugated with MWCNTs via π-π interaction to produce nAg-MWCNTs and then mixed with silver flakes (Fig. 7b, d). The nAg-MWCNTs form an effective electrical network among the silver flakes, which results in a conductivity of as high as 5710 S/cm at 0 % strain and 20 S/cm at 140 % strain.

**Fig. 8:**
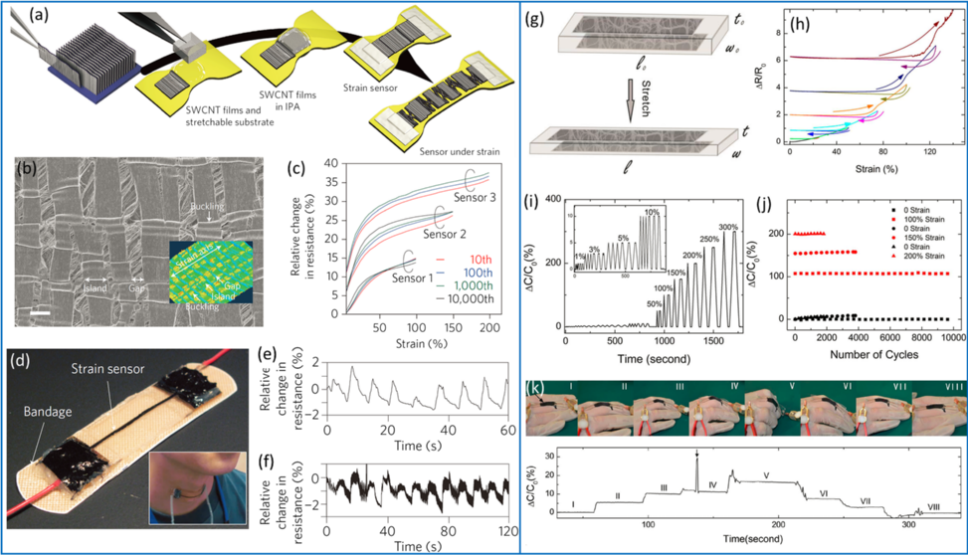
Stretchable resistive (**a**–**f**) and capacitive (**g**–**k**) strain gauges using CNT networks. **a** Schematics of the fabrication process and working mechanism of the resistive strain gauge. **b** In situ SEM image of the SWCNT array under a 100 % strain, showing the transverse fracture of the film. **c** Relative change in resistance for up to 10,000 stretching cycles with strains of 100, 150, and 200 %. **d**–**f** A strain gauge attached to a bandage and adhered to the throat (**d**) used to detect human breathing (**e**) and phonation (**f**). Reproduced from ref. [29]. **g** Operating mechanism of capacitive strain gauge using SWCNT film as stretchable electrodes. **h** Relative changes in resistance of the SWCNT/PDMS composite electrodes under progressively increasing strains. **i** Relative changes in capacitance under stretch-release cycles with progressively increasing strains. **j** Relative changes in capacitance after repeated stretch-release cycles with maximum strain of 100, 150, and 200 %. **k** Demonstration of using the capacitive strain gauge to monitor the motion of human fingers. *Top panel*: photographs of a strain gauge attached to the finger with different gestures. *Bottom panel*: The capacitive response at each corresponding stage. Reproduced from ref. [84]

Thanks to the above progress in carbon nanotube stretchable conductors, several stretchable functional electronic devices have been demonstrated, including strain gauge [29–31], pressure sensor [22], organic light emitting diode (OLED) [33, 86], and supercapacitor [34–36]. In particular, such nanomaterial-based stretchable strain gauges can detect very large strains (typically >30 %) that are far beyond the limit of conventional metal foil or semiconductor strain gauges. In addition, the unique mechanical compliance renders them suitable for the applications involving interfacing with biological tissue such as human motion detection, health monitoring, and rehabilitation. Figure 8 presents two examples of carbon nanotube stretchable strain gauges that are used for human motion detection. Yamada et al*.* demonstrated a resistive strain gauge (Fig. 8a–f) that explores the lateral fracture of as-grown SWCNT arrays, where the controlled opening and closing of cracks lead to reproducible resistive response upon repetitive stretching and releasing [29]. The device can detect strains as high as 280 % with very small amount of overshoot and relaxation. In another study, Cai et al*.* reported a capacitive strain gauge (Fig. 8g–k), which is assembled into a parallel-plate capacitor using two layers of CVD-grown carbon nanotube thin films as stretchable electrodes and a piece of silicone elastomer as the dielectric layer [31]. When stretched, the Poisson deformation of the elastomer in the device results in an increase of capacitance, which is found to be proportional to the applied strain. Due to the excellent stretchability of the carbon nanotube/silicone composite electrodes, the strain gauge exhibits stable and reliable capacitive response throughout the course of repetitive stretching with a maximum strain of up to 300 %.

**Fig. 9:**
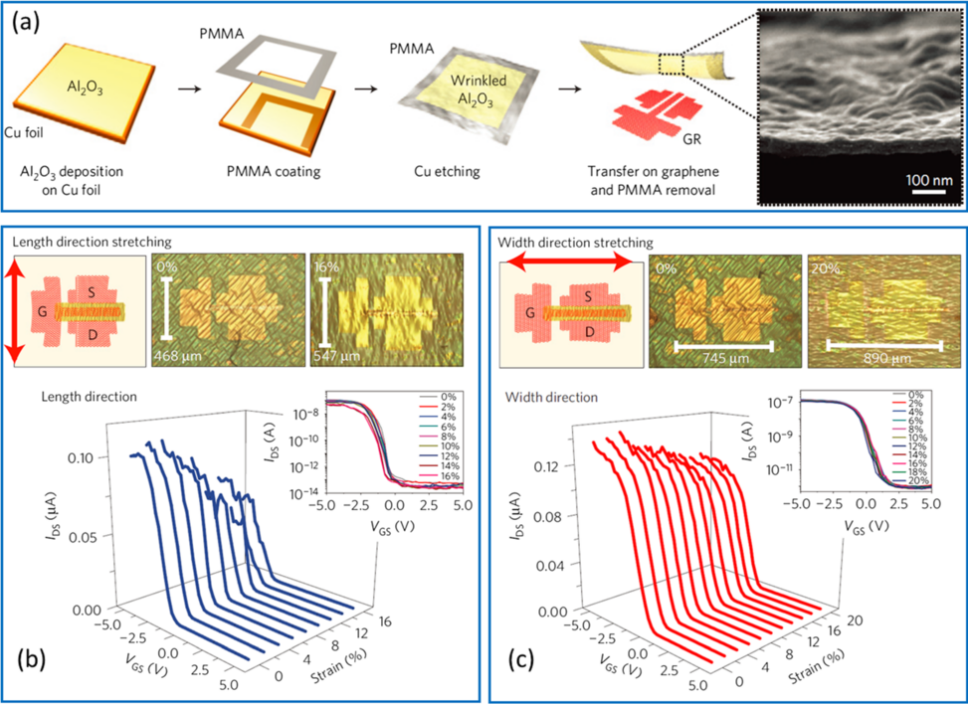
Stretchable CNT TFTs enabled by wrinkled Al_2_O_3_ gate dielectrics. **a** Deposition and transfer of Al_2_O_3_, which is randomly wrinkled during the transfer process. The SEM image shows the surface morphology of the wrinkled Al_2_O_3_. **b, c** Schematics, optical micrographs, and transfer characteristics of the fully fabricated CNT TFTs when stretched along the channel length direction up to a strain of 16 % (**b**) or along the channel width direction up to a strain of 20 % (**c**). Reproduced from ref. [27]

**Fig. 10:**
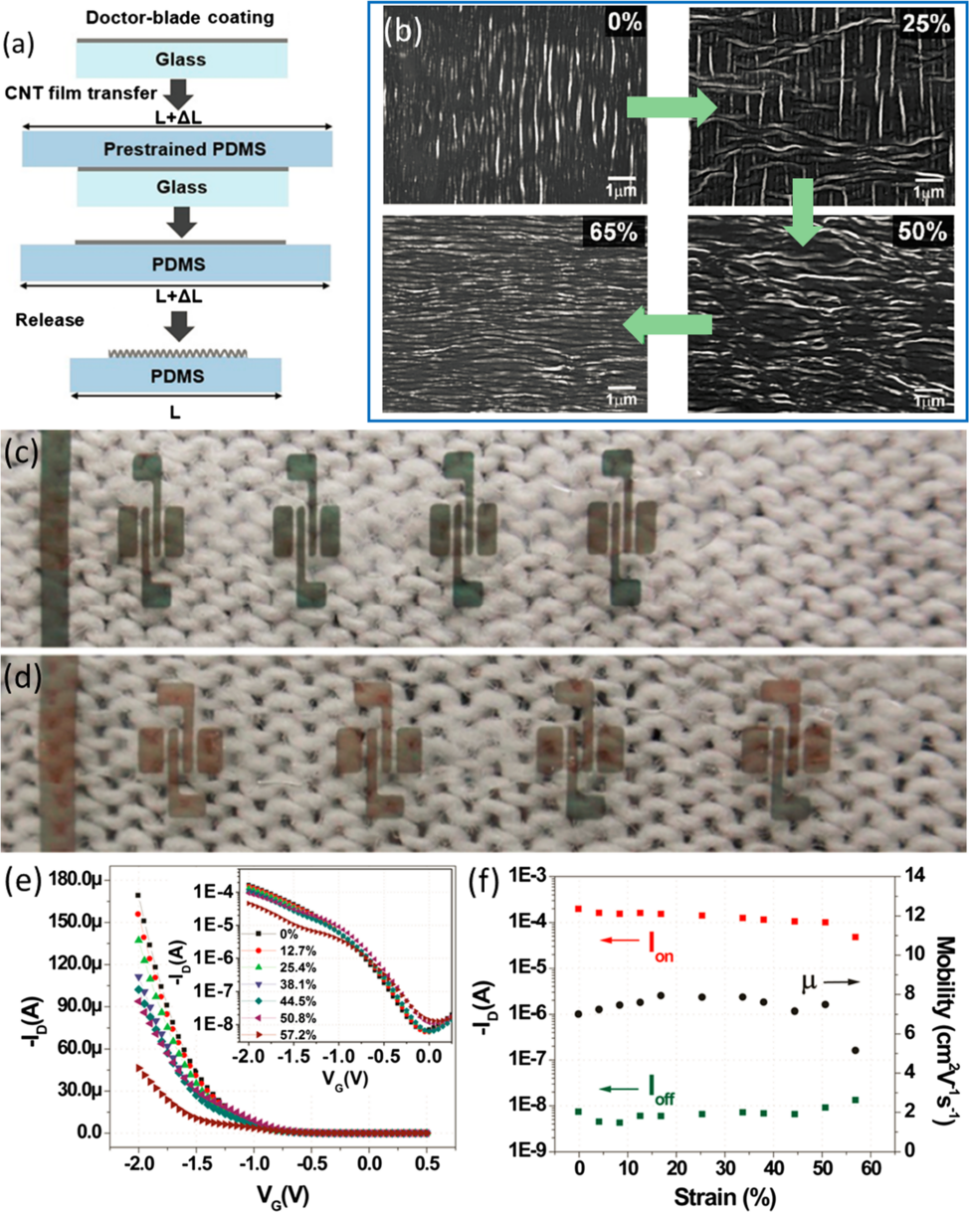
Intrinsically stretchable CNT-TFTs. **a** Schematics of the fabrication process where CNT film is transferred to prestrained (50 %) PDMS substrate, forming buckled structure after the prestrain in PDMS is released. **b** In situ SEM images of the buckled CNT film obtained after prestretched to 25, 50, and 65 %. **c, d** Photographs of the TFTs before (**c**) and after (**d**) stretched to 30 %. **e, f** Stretching test showing the transfer characteristics (**e**), mobility, on-current, and off-current (**f**) of the CNT-TFTs at various tensile strains up to 57.2 %. Reproduced from ref. [28]

To date, most of the research effort has been focused on stretchable conductors whereas the potential of using semiconducting SWCNTs as channel materials for stretchable transistors is rarely explored. The hurdles of realizing stretchable transistors are manifold. One bottleneck is the gate dielectric material, which is usually brittle. A well-known strategy to make conventional inorganic material stretchable is to induce buckles or wrinkles in the thin films [74]. Chae et al. report that randomly distributed wrinkles can be formed in Al_2_O_3_ thin film during a transfer process, imparting the film a certain degree of stretchability (Fig. 9) [27]. The wrinkled Al_2_O_3_ is then used as the gate dielectric layer for sSWCNT transistors, which exhibit stable electrical characteristics under a tensile strain up to 20 %. Another approach is to exploit intrinsically stretchable dielectric materials such as polymer electrolyte, ionic liquid, or ion gel [87]. The working mechanism of such type of dielectric materials is that, upon a positive (negative) gate bias voltage, the cations (anions) in the dielectric migrate towards and accumulate at the dielectric/semiconductor interface, which electrostatically induce the accumulation of electrons (holes) in the semiconductor [87]. Xu et al*.* fabricated stretchable SWCNT transistors using ion gel as the dielectric material and achieved a stretchability of over 50 %, which is the highest value for stretchable SWCNT transistors reported so far (Fig. 10) [28].

In addition to the selection of suitable dielectric material, stable interface between different components also plays a critical role as minute interfacial sliding or delamination may lead to catastrophic failure of the entire device. However, the huge discrepancy in the mechanical and chemical properties of different components poses a great challenge in acquiring a stable interface that can survive large strains (>100 %) [73]. For instance, the modulus of SWCNTs can reach 1 TPa [14], more than six orders of magnitude higher than that of common elastomer substrates like PDMS. As a result, the realization of highly stretchable and robust SWCNT transistors still needs intensive research efforts in material synthesis, structure design, and fabrication techniques.

**Fig. 11:**
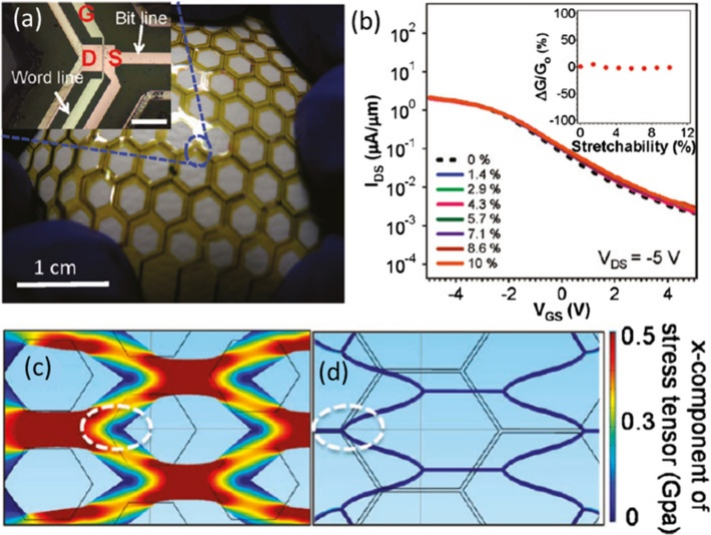
Structurally stretchable CNT-TFT backplane enabled by honeycomb mesh structure. **a** Photograph of the CNT-TFT backplane conformally covering a baseball. Inset shows the micrograph of one transistor. **b** Transfer characteristics measured at various tensile strains up to 10 %. **c, d** Mechanical simulation of hexagonal mesh structures with side lengths of 1.25 (**c**) and 1.85 mm (**d**) when stretched by 2 mm in horizontal direction. The *white dashed circles* mark the locations of active devices. Reproduced from ref. [68]

Furthermore, it is still possible to achieve stretchable electronic systems even if the various materials used are not intrinsically stretchable. For example, in another study, Takahashi et al*.* reported a conformal pressure sensor array that consists of a stretchable SWCNT active-matrix backplane (Fig. 11) [68]. Despite the fact that both the metal interconnection and ceramic dielectric layer materials are not stretchable, the backplane is rendered stretchable by laser cutting the substrate into a honeycomb mesh structure and strategically placing the transistors at the corners where the strain is minimized. This work represents yet another viable path to integrate SWCNT transistors into stretchable functional electronic systems.

### Scalable Fabrication Process—Printing

Printing as a new manufacturing method for macroelectronics is attracting a great deal of research interests due to the promise of high-speed and large-scale production of electronic devices in novel form factors [55, 56]. Because no photolithographic patterning or vacuum-based deposition/etching equipment is needed, the cost of electronic devices can be substantially reduced. Additionally, printed electronics can be fabricated on many unconventional substrates, including plastics, papers, textiles, and even rubbers, thus enabling numerous beyond-silicon applications ranging from smart packaging to electronic paper and from large-area smart wallpaper to ubiquitous wearable electronics [2]. Organic semiconductors have been the mainstay in printed electronics for a long time [5], whereas nanomaterials, among which are quantum dots [7], nanotubes [12], nanowires [88], and two-dimensional (2D) nanomaterials [10, 11], are new members of printable functional inks. In fact, many nanomaterials have already significantly outperformed organic semiconductors. For example, the field-effect mobility of printed TFTs using sSWCNTs is generally higher than 1 cm^2^V^−1^ s^−1^, which marks the upper limit for organic semiconductors. So far, several printing methods have been used to fabricate SWCNT-based devices, such as ink-jet printing [45–47, 59, 65], screen printing [58], and gravure printing [60–63, 66]. In the following section, we summarize the state-of-the-art development of printed electronics using carbon nanotubes. Although there are several other studies of printed electrical interconnects based on carbon nanotubes [81, 80], we focus on the use of carbon nanotubes as channel semiconductors though.

**Fig. 12:**
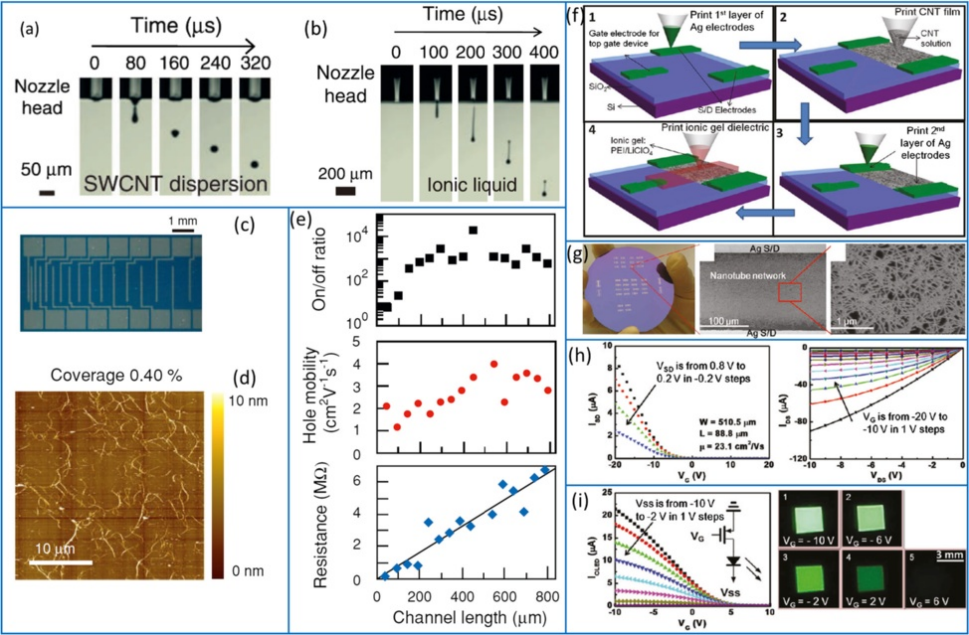
Ink-jet-printed SWCNT TFTs. **a, b** Snapshots of DMF-based SWCNT ink droplet for channel semiconductor (**a**) and ionic liquid droplet for gate dielectric (**b**) during the ink-jet printing process. **c** Optical micrograph of ink-jet-printed TFT array with gold contact electrodes. **d** AFM image of the printed SWCNT network in the channel of the TFT. **e** Off/off ratio (*top*), hole mobility (*middle*), and channel resistance (*bottom*) as functions of channel length. Reproduced from ref. [45]. **f** Schematics of fabricating polymer electrolyte gated SWCNT TFTs by an ink-jet-like process. **g** Photograph and optical micrograph of fully printed SWCNT TFTs. The SEM image on the *right* shows the SWCNT network with a high density. **h** Representative transfer (*left*) and output (*right*) characteristics of the fully printed SWCNT TFTs. **i** Application of printed CNT-TFT in an OLED control circuit. Reproduced from ref. [47]

Okimoto et al*.* pioneered the area of ink-jet-printed SWCNT TFTs by using the same SWCNT material for both contact electrodes and channel semiconductors (Fig. 12a–e) [45]. SWCNT networks with low density are used as the channel of the TFT, while more printing runs lead to a higher nanotube density which can be used as the contacts. They also proposed the utility of ionic liquid as gate dielectric layer to enable the top-gate operation. However, because the ink material is a mixture of metallic and semiconducting nanotubes, very sparse SWCNT network is required for achieving decent on/off current ratio, which unavoidably leads to rather low drive current. Using pre-sorted semiconductor-enriched SWCNTs can lead to devices with both high on/off ratio and large output current. Chen et al*.* demonstrated high-performance back-gated sSWCNT TFTs using an ink-jet-like printing method (Fig. 12f–h) [47]. The on/off ratio, on-current, and mobility reach 10^4^, 18 μA/mm (*V*_ds_ = −0.8 V), and 23 cm^2^V^−1^ s^−1^, respectively. They further demonstrated a single-pixel OLED control circuit using such fully printed nanotube TFTs (Fig. 12i).

Gate dielectric layer plays a crucial role and poses a major challenge in printing top-gated TFTs on flexible substrates. The widely adopted printable dielectrics are polymers which have rather low dielectric constant, resulting in a large operating voltage. In addition, it is very difficult to get an ultrathin dielectric film with good uniformity and clean interface through printing processes; any pinhole or interfacial trap would lead to poor device characteristics. Besides, the realization of flexible TFTs requires the dielectric material to be highly compliant, posing a greater challenge in selecting suitable materials. Two of the promising printable dielectric material platforms that have been studied extensively are ion gels [46, 59] and organic-inorganic hybrid dielectrics [58, 60, 61, 66]. Due to the formation of electrical double layers at the gate/dielectric and dielectric/semiconductor interfaces, ion gel offers very large gate capacitance, which is, in principle, independent of the film thickness [87]. Hybrid dielectrics consisting of polymers and inorganic nanoparticles are also excellent platforms for printed and mechanically compliant TFTs because they not only possess high dielectric constant and mechanical flexibility/stretchability but could also enable low-cost and scalable solution-based processing [89].

**Fig. 13:**
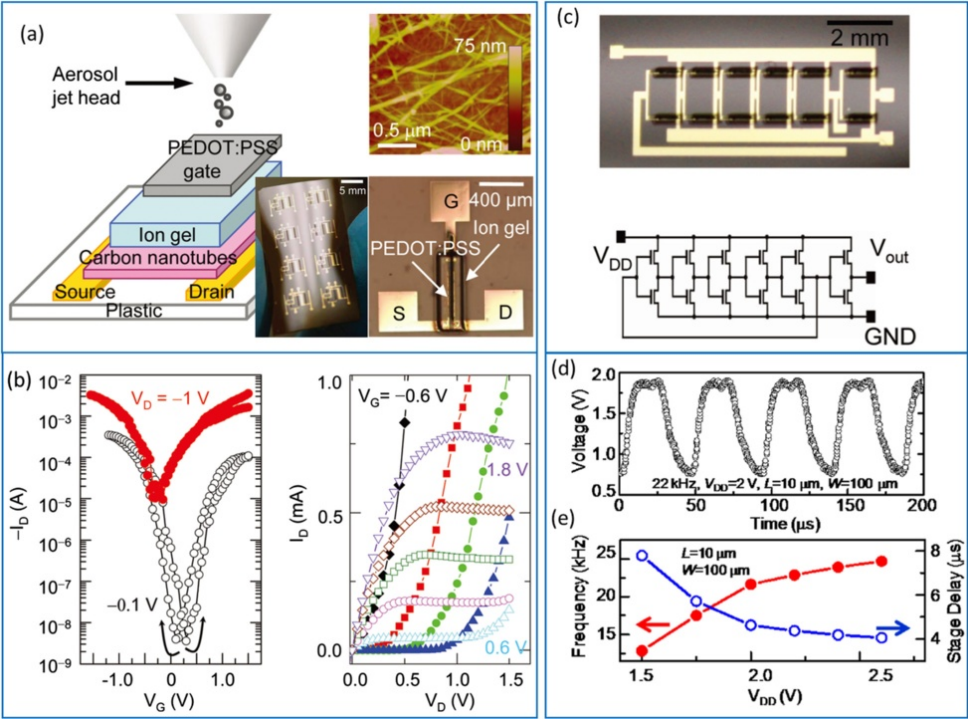
Printed CNT-TFT and integrated circuits using ion-gel dielectrics. **a** Schematic diagram and optical micrograph of the printed CNT TFT on flexible substrate with ion-gel dielectric and PEDOT:PSS gate electrode. The *top right* AFM image shows the printed CNT network. **b** Representative transfer (*left*) and output (*right*) characteristics of the printed TFT. **c** Photograph and circuit schematic of a ring oscillator with printed TFTs. Reproduced from ref. [46]. **d** Output signal of a ring oscillator driven by a supply voltage of 2 V with a stage delay time of 4.5 μs. **e** Frequency and stage delay times as functions of supply voltage. Reproduced from ref. [59]

Ha et al*.* pioneered the field of ionic-gel-gated sSWCNT TFTs by using an aerosol jet printing process (Fig. 13a, b) [46]. Gold source/drain and gate electrode arrays are first patterned by conventional photolithography and lift-off processes. Next, semiconducting SWCNTs, ion gel dielectrics, and PEDOT:PSS gate electrodes are printed successively into the channel regions, resulting in flexible TFTs with high hole and electron mobility of 31 and 17 cm^2^V^−1^ s^−1^, respectively. With ion gel dielectrics, the gate operating voltage of the transistors can be very low (<3 V) because of the huge gate capacitance (>1 μF/cm^2^). However, due to the small drifting velocity of ions, the switching speed is much lower than conventional dielectrics, which needs further improvement in the future. Nonetheless, recently, the same group demonstrated partially printed five-stage ring oscillators with stage delays lower than 5 μs at supply voltages below 3 V, representing an inspiring achievement towards high-performance printed circuits using carbon nanotubes (Fig. 13c–e) [59].

**Fig. 14:**
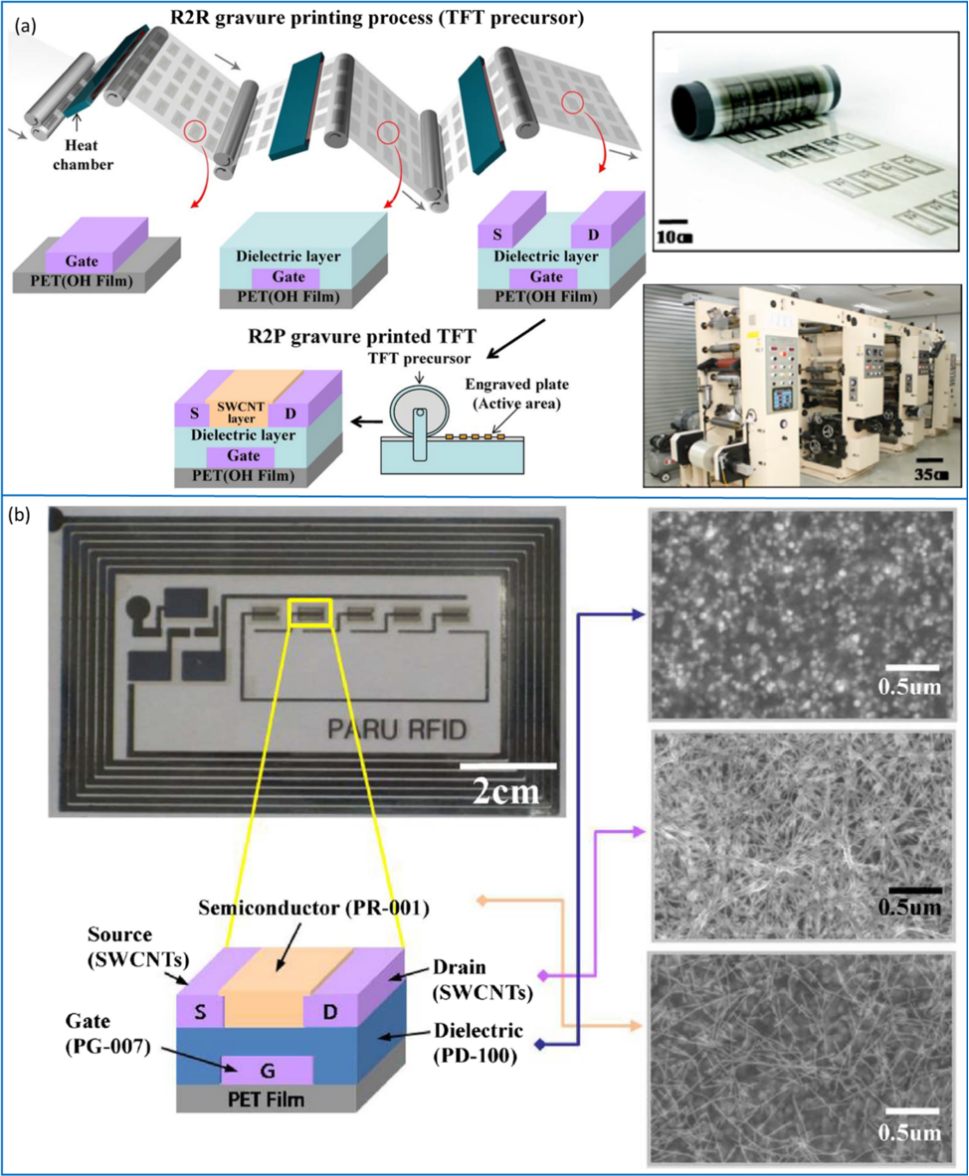
Flexible CNT-TFTs and integrated circuits fabricated using a roll-to-roll gravure printing process. **a** *Left*: schematics illustrating printing process for the fabrication of CNT-TFTs. *Upper right*: photograph of roll-to-roll printed 13.56 MHz RFID tags. *Lower right*: photograph of the gravure printer system. Reproduced from ref. [62]. **b** Optical picture of the printed CNT-TFTs in the 1-bit RFID tag and SEM images of the various components in the transistor after printing. Reproduced from ref. [60]

**Fig. 15:**
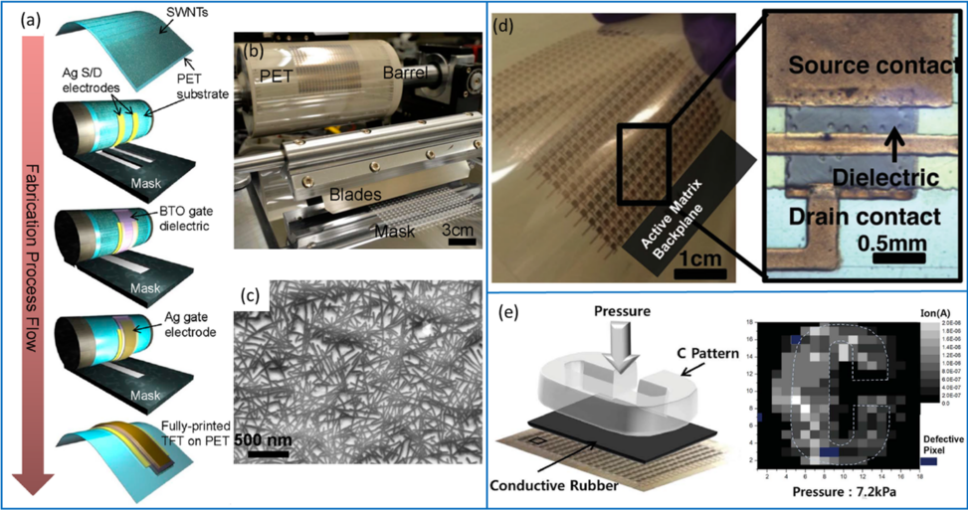
CNT-TFTs fabricated using a roll-to-plate printing process for applications in E-skin. **a** Schematics of the simplified gravure printing process (roll-to-plate). **b** Photograph of the roll-to-plate printer. **c** SEM image of the printed CNT network in the channel region of the TFT. Reproduced from ref. [61]. **d** Printed flexible active-matrix backplane for E-skin application. Inset: Enlarged view showing a single printed CNT-TFT. **e** Application of the printed active-matrix backplane for E-skin to map the applied pressure profile. Reproduced from ref. [66]

Compared with ink-jet printing, gravure printing (roll-to-roll or roll-to-plate) is a more cost-effective way for mass production of electronic devices over large areas. In a series of work by Paru Inc. in South Korea, researchers have demonstrated fully printed logic gates, half adder, D-flip-flop, and one-bit radio-frequency identification (FRID) tags through a gravure printing process (Fig. 14) [60, 62, 63]. In these studies, the silver gate electrodes, BaTiO_3_/PMMA hybrid dielectrics, semiconducting SWCNTs, and silver source/drain electrodes are sequentially printed onto flexible PET substrates in a roll-to-roll or roll-to-plate manner. For such gravure printing processes, the viscosity and surface tension of the inks and surface chemistry of the substrates are critical and need to be optimized for uniform and reproducible device performance. Beside digital integrated circuit applications, gravure-printed SWCNT TFTs have also been incorporated in functional electronic systems such as skin-like sensor arrays. Very recently, Yeom et al. reported the utility of gravure-printed nanotube TFT arrays as active-matrix backplanes for driving large-area-compliant tactile sensor arrays (Fig. 15) [66]. The active-matrix backplane consists of up to 400 TFTs with high yield (97 %) and excellent uniformity. Special attention should be paid to the overlap regions between the column and row selection lines to avoid leakage current between them. The system is capable of detecting pressures ranging from 1 to 20 kPa with a linear sensitivity of 800 %/kPa and spatially mapping the pressure profile (Fig. 15e). In addition, owing to the mechanical compliance of the hybrid dielectrics, the active-matrix backplane is highly flexible with invariant electrical characteristics when bent to a curvature radius of 1.85 cm.

**Fig. 16:**
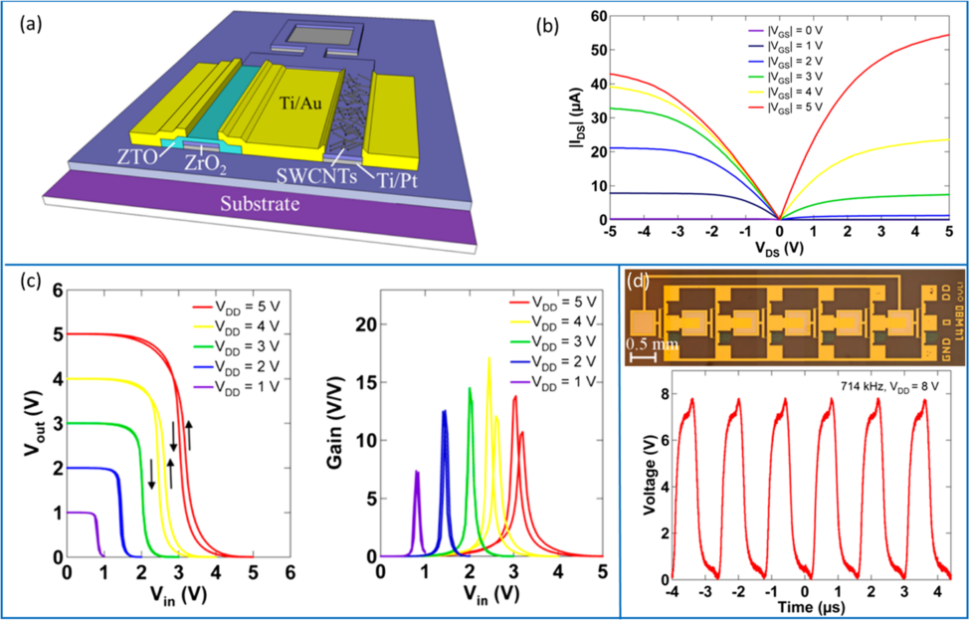
Heterogeneous integration of CNT (p-type) and ZTO (n-type) semiconductors for printed complementary integrated circuits. **a** Schematic diagram of the CMOS inverter. **b** Output characteristics of the printed n-type and p-type TFT. **c** VTC (*left*) and voltage gain (*right*) of the printed inverter under various supply voltages. **d** Printed 5-stage ring oscillator using the complementary inverters. *Top*: optical micrograph. *Bottom*: output signal of the oscillator driven by a *V*_dd_ of 8 V. Reproduced from ref. [94]

Complementary metal-oxide semiconductor (CMOS) operation is desired for digital logic applications as it provides rail-to-rail swing, large noise margin, and small static power consumption [12]. However, as-grown carbon nanotubes typically exhibit p-type behavior in ambient air due to lower Schottky barriers for holes than electrons at the nanotube/metal interfaces for most air-stable metal contacts. Despite of the numerous efforts [90–92], acquiring air-stable and reproducible n-type carbon nanotube transistors in a facile way remains challenging. Alternatively, the heterogeneous integration of p-type carbon nanotube and n-type metal oxide transistors is a promising strategy to realize complementary macroelectronic circuits [93]. Many metal oxide semiconductors, including zinc tin oxide (ZTO) and indium zinc oxide (IZO), can be solution processed, thereby opening the possibility of fully printed hybrid CMOS integrated circuits consisting of p-type SWCNTs and n-type metal oxides [94, 95]. Recently, Kim et al*.* reported high-speed, ink-jet printed SWCNT/ZTO hybrid complementary ring oscillators with stage delay of 140 ns, which represents the fastest ring oscillator with printed semiconductors to date (Fig. 16) [94]. In this study, gate and source/drain electrodes are defined by photolithography and lift-off processes and a double layer of ZrO_2_ is deposited by a sol-gel route as the gate dielectric. SWCNTs and ZTO are deposited from solutions using an ink-jet printer. This work indicates that hybrid complementary configuration is a useful approach for achieving fully printed and sophisticated macroelectronics by circumventing the difficulty in obtaining n-type SWCNT transistors.

## Conclusions

Tremendous progress has been made in SWCNT-based flexible and stretchable electronics. Nonetheless, almost no SWCNT-based flexible electronic product is commercially available at this moment [14]. Several challenges remain to be overcome before SWCNT-based electronic devices and systems can be made ready for the applications in consumer markets.

In the material aspect, although semiconductor-enriched SWCNTs are already commercially available in large quantities, there is still large inhomogeneity in terms of chirality and nanotube length. It is expected that high purity and good homogeneity of the starting material is beneficial for uniform device performance. Additionally, longer nanotubes are desired to reduce the number of tube-to-tube junctions, which could lead to further improvement in device mobility. However, the dissolution and separation of long nanotubes (>10 μm) are not easy. Furthermore, the effects of surfactants on device electrical characteristics require more thorough investigation. The surfactants used to disperse SWCNTs are difficult to remove and can act as barriers for electronic conduction, thus increase the contact and channel resistance. In the future, new surfactant-free methods need to be explored to effectively dissolve SWCNTs without damaging or shortening them. Recent studies of dispersing SWCNTs using superacids or salt-ammonia solutions show promise in this direction [96, 97]. Other problems confronting researchers include methods to obtain air-stable n-type conduction in SWCNTs and improve the uniformity, yield, and stability of SWCNT-based devices.

At the fabrication process end, although printing has been demonstrated to be a promising method for large-scale and low-cost manufacturing, the printed devices are still far inferior to their counterparts fabricated using conventional microfabrication processes in terms of electrical performance and uniformity. This is mainly caused by the low resolution (typically >50 μm) and poor reproducibility of current printing methods. Future work on printed SWCNT devices should also focus on improving the metal contacts and developing new dielectric materials. Stretchable electronics are relatively new and have attracted significant research interests. Despite the excellent stretchability of both sSWCNT networks and SWCNT thin-film electrodes, the realization of compliant dielectrics and robust interfaces appears to be the bottleneck in this field. Furthermore, fully printed stretchable systems have yet to be realized. Finally, graphene and other 2D semiconducting materials are also showing increasing potential for flexible/stretchable electronics [10, 11, 98, 99]. Incorporating SWCNTs with other forms of nanomaterials may lead to some exciting results [27].
